# Associations of length of stay with sarcopenia parameters in geriatric patients: age and sex differences in the oldest old

**DOI:** 10.1007/s40520-026-03442-z

**Published:** 2026-06-25

**Authors:** Konstantinos Prokopidis, Hanne Nygaard, Rikke S Kamper, Pernille Hansen, Sofie K Hansen, Eckart Pressel, Jens Rasmussen, Charlotte Suetta

**Affiliations:** 1 Department of Musculoskeletal and Ageing Science, Institute of Life Course and Medical Sciences, University of Liverpool, 6 West Derby St, Liverpool, L7 8TX UK; 2 Department of Emergency Medicine, Copenhagen University Hospital, Bispebjerg and Frederiksberg, Copenhagen, Denmark; 3 Geriatric Research Unit, Department of Geriatric and Palliative Medicine, Copenhagen University Hospital, Bispebjerg and Frederiksberg, Copenhagen, Denmark; 4 Faculty of Health and Medical Sciences, Institute of Clinical Medicine, CopenAge - Copenhagen Center for Clinical Age Research, University of Copenhagen, Copenhagen, Denmark; 5 Department of Clinical Medicine, Faculty of Health, University of Copenhagen, Copenhagen, Denmark

**Keywords:** Sarcopenia, Length of stay, Inpatients, Physical function, Frailty

## Abstract

**Background:**

Hospital-associated deconditioning is a contributor to secondary sarcopenia, which may be driven by prolonged hospital length of stay (LoS). This study investigated the bidirectional association of LoS and sarcopenia indices (lean soft tissue, muscle strength, physical function) in older inpatients, with a focus on age- and sex-specific differences.

**Methods:**

Using cross-sectional data from the Copenhagen PROTECT study, 1071 older inpatients (aged ≥ 65 years) were included. Appendicular lean soft tissue index (ALSTI), handgrip strength (HGS), gait speed, and 30-second chair stand test (30CST) were assessed within 24 h of admission and sarcopenia was defined per the EWGSOP2 criteria. Linear and logistic regressions examined associations between LoS and sarcopenia indices, stratified by age and sex.

**Results:**

In patients ≥ 80 years, longer LoS (based on the cohort’s median) was associated with lower gait speed and 30CST in both sexes (*p* < 0.01), and lower HGS in women (*p* = 0.03). In men < 80 years, longer LoS correlated with lower HGS and 30CST (*p* < 0.01), with higher odds of sarcopenia (Low ALSTI and low 30CST; OR = 3.14, *p* = 0.03). Higher 30CST and HGS were consistently associated with shorter LoS, particularly in men ≥ 80 years and women across both age groups.

**Conclusions:**

HGS and 30CST are strongly associated with LoS in older inpatients, with more pronounced effects in those ≥ 80 years. Future longitudinal studies and trials are warranted to confirm causality, that may guide rehabilitation strategies to reduce LoS through targeted interventions.

**Supplementary Information:**

The online version contains supplementary material available at https://doi.org/10.1007/s40520-026-03442-z.

## Introduction

In older age, hospitalization may lead to significant declines in muscle mass, strength, and physical function, potentiating a process known as hospital-associated deconditioning [1]. This phenomenon is a contributor to secondary sarcopenia and is driven by immobilisation, systemic inflammation, and burdening of the respective condition(s) [2]. The duration of hospital length of stay (LoS) plays a key role in exacerbating these effects. For instance, prolonged LoS may promote muscle atrophy and functional decline due to increasing sedentary activity and/or immobilization, posing substantial risks for older inpatients [3], including increased dependence [4], frailty [5], and higher rates of adverse outcomes such as falls and rehospitalization [6]. These risks are particularly pronounced in the older age, where age-related physiological changes amplify vulnerability to impaired muscle health and physical function, as well as reconditioning [7–10]. Previous research in older adults has shown a reduction in rate of force development of the lower limb, following 5 days of bed rest [11], while Coker et al. (2015) also showed significant decreases in appendicular lean soft tissue, muscle quality, chair stand test, gait speed, and 5-minute walk distance [12]. A recent meta-analysis [3] demonstrated a negative impact from increasing LoS on handgrip strength (HGS), with an overall pooled incidence of 18% of acute sarcopenia in hospitalized populations, for which, 59% were already admitted in intensive care units (ICU). However, several measures of physical function, including 6-minute walk, timed-up-and-go, and gait speed, remained unchanged, reflecting patient recovery from potentially acute conditions that initially led to hospitalization, partly compensating for hospital-associated deconditioning in patients with severely impaired function at baseline. Additionally, this study identified age as a significant predictor of timed-up-and-go performance, with older age being associated with reduced performance. In relation to this, in community-dwelling adults, reductions in quadriceps power/kg has been approximately 1.03% per year from the sixth to seventh decade of life, 1.42% per year from the seventh to eighth decade of life, and 2.36% per year from the eighth to ninth decade of life [13]. This suggests that distinct impairments exist among the oldest old who may be more vulnerable, considering that increasing age could overlap with higher comorbidity rates, frailty, dependency, sarcopenia, and even LoS in older inpatients [14, 15]. In addition, muscle pathologies may affect differently men and women, in part due to muscle protein turnover, satellite cell content and proliferation, hormonal interactions, and mitochondrial differences [16]. Whether there is a link between LoS duration and measures of sarcopenia among older adults and between sexes, is yet to be elucidated. Using cross-sectional data from the Copenhagen PROTECT study, we aimed to explore the bidirectional association of different LoS durations with baseline measurements of appendicular lean soft tissue (ALST), muscle strength, and physical function, and whether these associations were stronger in the oldest old (> 80 years) and are sex-specific.

## Methods

### Subjects and study design

This study utilizes secondary, cross-sectional data from the Copenhagen PROTECT cohort, a prospective study that has registered at Clinicaltrials.gov (NCT04151108). The current analysis utilized data from 1071 older inpatients (aged ≥ 65 years) acutely admitted to Copenhagen University Hospital, Bispebjerg and Frederiksberg, from November 2019 to November 2021. All patient testing was conducted within 24 h of admission, per the study protocol [17]. Written informed consent was obtained from all participants or, in cases of persistent or fluctuating cognitive impairment, from relatives in collaboration with an independent physician. The study adhered to the Declaration of Helsinki and was approved by the local ethics committee of Copenhagen and Frederiksberg (H-19039214).

### Body composition, muscle strength, and physical function assessments

ALST was assessed via direct segmental multifrequency bioelectrical impedance analyses (DSM-BIA) (Inbody S10; BioSpace Co., Ltd, Seoul, South Korea). ALST adjusted for height squared (ALST/h^2^) is known as appendicular lean soft tissue index (ALSTI). Similarly, total body-, intracellular-, and extracellular water were measured. Muscle strength was evaluated through HGS using a digital handheld dynamometer (Model SH1001; SAEHAN Corporation, Yangdeok-Dong, Masan, South Korea), for which, the highest measurement from three attempts of the dominant hand was recorded as the outcome [17]. Gait speed was measured using patients’ habitual speed over a 4-meter distance, in which, the time from the start to the finish line was recorded [17]. Thirty-second chair stand test (30CST) was employed to assess the number of repetitions the participants could perform within 30 s [17]. When outcomes were treated categorically, the median value of the corresponding population of interest (≥ 80 years vs. <80 years of age) was selected to classify total body-, intracellular-, and extracellular water as “higher”.

### Definition of sarcopenia

Sarcopenia was defined based on: (1) ALSTI below < 7.0 kg/m^2^ for men and < 5.5 kg/m^2^ for women, plus a HGS below < 27 kg for men and < 16 kg for women, or (2) ALSTI below < 7.0 kg/m^2^ for men and < 5.5 kg/m^2^ for women, plus a 30CST < 8 repetitions, in accordance with the European Working Group on Sarcopenia in Older People (EWGSOP2) [18].

### Demographic and clinical data

Body mass index was measured using patients’ bodyweight divided by height squared (kg/m^2^). Hospital medical records were used to determine the number of hospitalizations during the last 12 months, readmission rates, days to readmission, living conditions (living alone or cohabiting), place the patients was discharged to and place following discharge, whether they were admitted to a geriatric department, all-cause and in-hospital death within 90 days from admission, age-adjusted Charlson Comorbidity Index [19], diagnosis categories related to index admission (previously shown [20] in Appendix 1), Early Warning Score (a total score based on respiratory rate, heart rate, blood pressure, oxygen saturation, temperature, and an assessment of consciousness) [21], number of medications, LoS, orientation, memory, while questionnaires were used relevant to the number of falls (as part of SARC-F questionnaire), being able to get out of bed, concentration (OMC) score, and clinical frailty scale (CFS) [22].

Information on results from standard blood samples drawn initially during the index admission was collected from medical history records. This information covered hemoglobin, creatinine, estimated glomerular filtration rate (eGFR), albumin, lactate dehydrogenase, glucose, and C-reactive protein. Early Warning Score and C-reactive protein are markers of disease severity [23]. Moreover, blood samples were collected from the antecubital vein within 24 h of hospital admission during patient admission. The blood was gathered in ethylenediamine tetraacetic acid-treated tubes, stored on ice, and then centrifuged at 2500 g at 4 °C for 10 min. Plasma was subsequently separated and stored at -80 °C until analysis. Assessments via R-PLEX electrochemiluminescence assays from MesoScale Diagnostics (MSD, Rockville, MD, USA) assisted with the concentrations of growth differentiation factor-15 (GDF-15), interleukin-6 (IL-6), IL-10, tumour necrosis factor-alpha (TNF-a), and transforming growth factor-beta 1 (TGF-b1).

### Statistical analysis

Descriptive statistics were calculated to determine relative percentages and mean, or median values based on the distribution type. The normality of data distribution was assessed using Kolmogorov-Smirnov tests. When data were normally and non-normally distributed, Mann Whitney-U tests and independent t-test, respectively, were performed. Chi-squared tests were performed for categorical variables. Furthermore, patient characteristics were classified based on age: 65-79.9 and ≥ 80 years. A subsequent comparison was made following sex-specific categorisation based on these two age groups. Continuous variables are presented as mean ± standard deviation (SD) and categorical variables as frequencies (percentage). Linear regressions were used to explore associations between longer LoS, defined as the median value of both age groups and sexes (4.42 days in males and 4.73 days in females) with ALSTI, ALST, HGS, gait speed, and 30CST. The median LoS value is closely related to our recent meta-analysis examining the impact of hospitalization on acute sarcopenia (median of 5.7 days) [3], and the mean value (5.8 days) of LoS of the entire cohort as we have previously shown [17]. Logistic regressions were conducted to explore links with those measures when they were treated as “higher” ALSTI, ALST, HGS, gait speed, and 30CST, as well as sarcopenia). Additionally, linear and logistic regressions were used with LoS and longer LoS, respectively, being treated as the dependent variable in the analyses. Higher ALSTI, ALST, HGS, gait speed, and 30CST were the independent variables. Logistic regressions estimated the odds ratios (OR) and 95% confidence intervals (95% CI). In terms of adjustments, we included an unadjusted model, a Model 1 adjusting for age and body mass index, and a Model 2 adjusting for Model 1 and Charlson Comorbidity Index. Significance was set at *P* < 0.05. All analyses were conducted using IBM SPSS Statistics version 30.0.0.

## Results

This study utilized data from patients enrolled in the Copenhagen PROTECT study (*n* = 1071). Patients aged 65 to 79.9 years had a sample size of 597 with a mean age of 73.0 (4.1) years and a body mass index of 26.0 (5.7) kg/m^2^. Patients aged 80 years and above consisted of 474 adults with a mean age of 86.1 (4.6) years and a body mass index of 25.4 (4.6) kg/m^2^. Details of both cohorts are shown in Table 1, while in Table 2, details of both cohorts are presented stratified by sex.

**Table 1:** Baseline hospital-related characteristics of acutely admitted older medical patients aged ≥ 80 years vs. <80 years of age. Data is expressed as mean (SD)

Outcomes	Patients ≥ 80 years	Patients < 80 years	*P* value
Sample size (men/women)	474 (190/284)	597 (313/284)	< 0.01*
Age (years)	86.1 (4.6)	73.0 (4.1)	< 0.01*
Body mass index (kg/m^2^)	25.4 (4.6)	26.0 (5.7)	0.53
Prior hospitalization(s) (in the last 12 months)	1.03 (1.72)	1.17 (1.76)	0.51
Readmission – n (%)	156 (32.9)	207 (34.7)	0.55
Time to readmission (days)	25.7 (22.3)	27.6 (24.1)	0.58
Living conditions – n (%) Cohabiting Living alone	127 (30.7) 286 (69.3)	213 (37.5) 354 (62.5)	0.03*
Place of Discharged to – n (%) Living independently Sheltered housing Nursing home	411 (86.9) 3 (0.06) 59 (12.7)	567 (95.0) 6 (0.1) 24 (4.0)	< 0.01*
Place after Discharge – n (%) Living independently Sheltered housing Nursing home Rehabilitation stay	329 (72.1) 1 (0.02) 67 (14.7) 59 (12.9)	478 (82.1) 6 (0.1) 26 (4.5) 72 (12.3)	< 0.01*
Admitted to geriatric department – n (%)	195 (41.2)	117 (19.6)	< 0.01*
Able to get out of bed – n (%)	384 (81.0)	495 (82.9)	0.37
All-cause death – n (%)	68 (14.3)	57 (9.5)	0.02*
In-hospital death – n (%)	17 (3.6)	16 (2.7)	0.39
Falls – n (%) None 1–3 4 or more	141 (43.7) 128 (39.6) 54 (16.7)	207 (46.2) 165 (36.8) 76 (17.0)	0.61
Charlson Comorbidity Index	5.9 (1.9)	4.8 (2.2)	< 0.01*
Early Warning Score	2.17 (2.26)	2.64 (2.54)	< 0.01*
Diagnosis categories related to index admission – n (%) Infectious diseases Haematological diseases Musculoskeletal diseases Respiratory system diseases Cardiovascular system diseases Digestive system diseases Nervous system diseases R-diagnoses Endocrine and nutritional diseases Mental and behavioural diagnoses Z-diagnoses Others	185 (39.1) 10 (2.1) 30 (6.3) 34 (7.2) 11 (2.3) 21 (4.4) 2 (0.4) 79 (16.7) 48 (10.1) 10 (2.1) 4 (0.8) 22 (4.7)	213 (36.9) 9 (1.6) 27 (4.7) 69 (12.0) 12 (2.0) 41 (7.1) 8 (1.4) 86 (14.9) 51 (8.8) 29 (5.0) 4 (0.7) 29 (5.0)	0.03*
No of medications	8.4 (4.0)	8.6 (4.7)	0.82
Length of stay (days)	6.73 (7.25)	6.76 (8.55)	0.41
Bodywater (kg)	32.08 (7.24)	35.08 (7.74)	< 0.01*
Intracellular water (kg)	19.04 (4.26)	21.04 (4.60)	< 0.01*
Extracellular water (kg)	13.04 (3.02)	14.04 (3.21)	< 0.01*
Body fat (kg)	24.5 (9.7)	25.1 (11.9)	0.98
Leg lean mass (kg)	13.01 (4.28)	14.64 (4.20)	< 0.01*
Arm lean mass (kg)	4.49 (1.44)	5.10 (1.74)	< 0.01*
Appendicular lean soft tissue (kg)	17.49 (5.48)	19.77 (5.61)	< 0.01*
Appendicular lean soft tissue index (kg/m^2^)	6.47 (1.40)	6.96 (1.50)	< 0.01*
Handgrip strength (kg)	18.9 (7.9)	23.9 (9.3)	< 0.01*
Gait speed (m/s)	0.62 (0.25)	0.61 (0.27)	0.98
30CST (repetitions)	4.01 (5.00)	5.92 (5.39)	< 0.01*
OMC score	19.6 (6.6)	21.3 (6.4)	< 0.01*
Clinical Frailty Scale	4.73 (1.31)	4.17 (1.37)	< 0.01*
Hemoglobin (mmol/l)	7.5 (1.3)	7.78 (1.42)	< 0.01*
Creatinine (µmol/l)	105.9 (71.3)	101.2 (76.8)	< 0.01*
eGFR (ml/min/1.73m^2^)	56.6 (21.3)	67.2 (23.0)	< 0.01*
Albumin (g/l)	31.1 (5.0)	31.3 (5.5)	0.21
Lactate dehydrogenase (u/l)	234.2 (149.1)	224.3 (115.7)	0.11
Fasting glucose (mmol/l)	7.55 (3.34)	7.71 (3.35)	0.88
C-reactive protein (mg/l)	61.3 (77.6)	68.2 (86.3)	0.26
GDF-15 (pg/ml)	4095.2 (4729.2)	3605.1 (4086.5)	< 0.01*
IL-6 (pg/ml)	23.29 (95.18)	21.21 (80.54)	0.83
IL-10 (pg/ml)	2.98 (13.31)	2.21 (8.16)	0.32
TNF-a (pg/ml)	3.49 (9.96)	2.62 (4.29)	< 0.01*
TGF-b1 (pg/ml)	8987.8 (6852.2)	10,005.8 (9701.5)	0.02*

*Indicates significance; *p* < 0.05

30CST, 30-second chair stand test; eGFR, estimated glomerular filtration rate; GDF-15, growth differentiation factor-15; IL, interleukin; OMC, Orientation, memory, and concentration; TGF-b1, transforming growth factor-beta 1; TNF-a, tumor necrosis factor-alpha

Patients ≥ 80 years; Body mass index → *n* = 315; Prior hospitalization → *n* = 470; Time to readmission → *n* = 156; Living conditions → *n* = 414; Place after Discharge → *n* = 456; LoS → *n* = 457; Handgrip strength → *n* = 458; Falls → *n* = 323; Body water → *n* = 258; Body fat → *n* = 258; Diagnosis → *n* = 473; Arm lean mass → *n* = 258; Leg lean mass → *n* = 257; ALST → *n* = 258; ALSTI → *n* = 258; Gait speed → *n* = 122; 30CST → *n* = 291; OMC → *n* = 427; Hemoglobin → *n* = 473; Creatinine → *n* = 473; eGFR → *n* = 473; Albumin → *n* = 468; Lactate dehydrogenase → *n* = 354; Fasting glucose → *n* = 459; C-reactive protein → *n* = 469; GDF-15 → *n* = 464; IL-6 → *n* = 464; IL-10 → *n* = 464; TNF-a → *n* = 464; TGF-b1 → *n* = 464; Bodywater → *n* = 258; Intracellular water → *n* = 258; Extracellular water → *n* = 258

Patients < 80 years; Body mass index → *n* = 419; Prior hospitalization → *n* = 589; Time to readmission → *n* = 207

Living conditions → *n* = 568; Place after Discharge → *n* = 582; LoS → *n* = 580; Handgrip strength → *n* = 572; Falls → *n* = 448; Body fat → *n* = 382; Body water → *n* = 382; Diagnosis → *n* = 577; Arm lean mass → *n* = 382; Leg lean mass → *n* = 382; ALST → *n* = 382; ALSTI → *n* = 382; Gait speed → *n* = 70; 30CST → *n* = 335; OMC → *n* = 549; Hemoglobin → *n* = 596; Creatinine → *n* = 592; eGFR → *n* = 593; Albumin → *n* = 582; Lactate dehydrogenase → *n* = 427; Fasting glucose → *n* = 571; C-reactive protein → *n* = 590; GDF-15 → *n* = 574; IL-6 → *n* = 574; IL-10 → *n* = 574; TNF-a → *n* = 574; TGF-b1 → *n* = 574; Bodywater → *n* = 382; Intracellular water → *n* = 382; Extracellular water → *n* = 382

**Table 2:** Sex-specific baseline hospital-related characteristics of acutely admitted older medical patients aged ≥ 80 years vs. <80 years of age. Data is expressed as mean (SD)

Outcomes	Men			Women		
	Patients ≥ 80 years	Patients < 80 years	*P* value	Patients ≥ 80 years	Patients < 80 years	*P* value
Sample size (n)	190	313		284	284	
Age (years)	85.4 (3.8)	72.9 (4.1)	< 0.01*	86.6 (5.0)	73.2 (4.1)	< 0.01*
Body mass index (kg/m^2^)	23.1 (7.6)	23.2 (9.7)	0.52	23.6 (8.5)	24.5 (8.2)	0.69
Prior hospitalization(s) (in the last 12 months)	1.11 (1.43)	1.24 (1.94)	0.81	0.98 (1.89)	1.08 (1.54)	0.65
Readmission – n (%)	78 (41.1)	120 (38.3)	0.55	78 (27.5)	87 (30.6)	0.41
Time to readmission (days)	23.0 (21.0)	27.7 (25.4)	0.44	28.3 (23.4)	27.4 (22.4)	0.94
Living conditions – n (%) Cohabiting Living alone	87 (50.9) 84 (49.1)	148 (49.7) 150 (50.3)	0.80	40 (16.5) 203 (83.5)	65 (24.1) 205 (75.9)	0.03*
Place of Discharged to – n (%) Living independently Sheltered housing Nursing home	171 (90.0) 0 (0) 19 (10)	297 (94.9) 4 (1.3) 12 (3.9)	< 0.01*	240 (84.5) 3 (1.1) 41 (14.4)	270 (95.1) 2 (0.7) 12 (4.2)	< 0.01*
Place after Discharge – n (%) Living independently Sheltered housing Nursing home Rehabilitation stay	137 (76.1) 1 (0.6) 22 (12.2) 20 (11.1)	246 (80.4) 4 (1.3) 14 (4.6) 42 (13.7)	0.02*	192 (69.6) 0 (0) 45 (16.3) 39 (14.1)	232 (83.5) 2 (0.7) 12 (4.3) 30 (10.8)	< 0.01*
Admitted to geriatric department – n (%)	83 (43.7)	55 (17.6)	< 0.01*	112 (39.4)	62 (21.8)	< 0.01*
Able to get out of bed – n (%)	153 (80.5)	255 (81.5)	0.80	230 (81.0)	240 (84.5)	0.27
All-cause death – n (%)	44 (23.2)	34 (10.9)	< 0.01*	24 (8.5)	23 (8.1)	0.88
In-hospital death – n (%)	9 (47.4)	8 (2.6)	0.19	8 (0.3)	8 (0.3)	1.00
Falls – n (%) None 1–3 4 or more	55 (42.0) 51 (38.9) 25 (19.1)	109 (46.6) 89 (38.0) 36 (15.4)	0.58	86 (45.0) 77 (40.3) 29 (15.2)	98 (45.8) 76 (35.5) 40 (18.7)	0.51
Charlson Comorbidity Index	6.47 (2.23)	5.14 (2.31)	< 0.01*	5.51 (1.60)	4.53 (1.92)	< 0.01*
Early Warning Score	2.28 (2.25)	2.65 (2.55)	0.13	2.09 (2.26)	2.64 (2.54)	< 0.01*
No of medications	7.8 (3.9)	8.5 (4.7)	0.21	8.8 (4.1)	8.7 (4.7)	0.61
Length of stay (days)	6.82 (7.44)	6.91 (9.40)	0.63	6.67 (7.14)	6.60 (7.52)	0.51
Body water (kg)	38.80 (5.88)	39.74 (6.92)	0.29	25.12 (10.58)	30.37 (5.29)	< 0.01*
Intracellular water (kg)	22.93 (3.46)	23.78 (4.11)	0.11	16.77 (2.80)	18.29 (3.22)	< 0.01*
Extracellular water (kg)	15.88 (2.48)	15.97 (2.91)	0.86	11.39 (1.87)	12.08 (2.16)	< 0.01*
Body fat (kg)	23.36 (8.02)	24.76 (11.70)		28.16 (4.60)	25.39 (12.17)	0.86
Appendicular lean soft tissue (kg)	22.62 (4.50)	23.27 (4.79)	0.25	15.51 (3.40)	16.23 (3.90)	< 0.01*
Appendicular lean soft tissue index (kg/m^2^)	7.49 (1.18)	7.65 (1.35)	0.39	5.87 (1.16)	6.26 (1.30)	< 0.01*
Handgrip strength (kg)	24.5 (7.7)	28.5 (9.3)	< 0.01*	15.2 (5.5)	19.0 (6.3)	< 0.01*
Gait speed (m/s)	0.67 (0.26)	0.63 (0.30)	0.45	0.58 (0.23)	0.62 (0.25)	0.56
30CST (repetitions)	4.2 (5.2)	6.1 (5.7)	< 0.01*	3.9 (4.9)	5.6 (5.2)	< 0.01*
OMC score	19.6 (6.0)	21.1 (6.6)	< 0.01*	19.6 (6.9)	21.5 (6.2)	< 0.01*
Clinical Frailty Scale	4.63 (1.32)	4.17 (1.40)	< 0.01*	4.80 (1.30)	4.18 (1.34)	< 0.01*
Hemoglobin (mmol/l)	7.5 (1.3)	7.8 (1.5)	< 0.01*	7.5 (1.2)	7.8 (1.3)	< 0.01*
Creatinine (µmol/l)	128.2 (92.8)	114.4 (85.7)	< 0.01*	90.9 (46.5)	86.7 (62.8)	< 0.01*
eGFR (ml/min/1.73m^2^)	54.9 (21.7)	66.3 (23.5)	< 0.01*	57.8 (20.9)	68.2 (22.5)	< 0.01*
Albumin (g/l)	30.2 (4.7)	30.9 (5.5)	0.06	31.7 (5.2)	31.8 (5.4)	0.61
Lactate dehydrogenase (u/l)	251.7 (178.8)	222.5 (122.9)	0.07	223.7 (127.2)	226.1 (108.1)	0.70
Fasting glucose (mmol/l)	7.76 (4.45)	8.00 (3.50)	0.18	7.41 (2.34)	7.39 (3.15)	0.17
C-reactive protein (mg/l)	68.4 (77.6)	72.4 (86.7)	0.96	56.6 (77.3)	63.7 (85.8)	0.32
Barthel Index	50.6 (28.4)	55.6 (34.9)	0.51	55.2 (29.6)	58.1 (33.6)	0.51
GDF-15 (pg/ml)	4995.3 (6592.9)	4147.8 (4731.0)	< 0.01*	3487.6 (2703.1)	2998.2 (3115.2)	< 0.01*
IL-6 (pg/ml)	24.28 (41.26)	20.51 (53.35)	0.15	22.62 (118.53)	21.99 (102.86)	0.45
IL-10 (pg/ml)	4.53 (20.01)	2.37 (10.25)	0.40	1.93 (4.96)	2.03 (4.87)	0.09
TNF-a (pg/ml)	3.24 (6.77)	2.54 (2.46)	0.09	3.65 (11.64)	2.72 (5.69)	< 0.01*
TGF-b1 (pg/ml)	7990.5 (5764.4)	9886.2 (10,937.8)	0.02*	9661.1 (7432.5)	10,139.6 (8117.4)	0.12

*Indicates significance; *p* < 0.05

30CST, 30-second chair stand test; eGFR, estimated glomerular filtration rate; GDF-15, growth differentiation factor-15; IL, interleukin; OMC, Orientation, memory, and concentration; TGF-b1, transforming growth factor-beta 1; TNF-a, tumor necrosis factor-alpha

Patients ≥ 80 years; Men; BMI → *n* = 133; HGS → *n* = 181; 30CST → *n* = 115; Gait speed → *n* = 49; Falls → *n* = 131; OMC → *n* = 164; Readmission → *n* = 190; Intracellular water → *n* = 95; Extracellular water → *n* = 95; Body water → *n* = 95; Bodyfat → *n* = 95; Appendicular Lean Soft Tissue → *n* = 95; Appendicular Lean Soft Tissue Index → *n* = 95; Length of Stay → *n* = 181; Time to Readmission → *n* = 78; Prior Hospitalization → *n* = 188; Albumin → *n* = 186; Lactate Dehydrogenase → *n* = 133; Glucose → *n* = 182; C-reactive protein → *n* = 189; Living conditions → *n* = 171; Place after Discharge → *n* = 180; Barthel Index → *n* = 48; GDF-15 → *n* = 187; IL-6 → *n* = 187; IL-10 → *n* = 187; TNF-a → *n* = 187; TGF-b1 → *n* = 187; *Women*; BMI → *n* = 209; HGS → *n* = 277; 30CST → *n* = 176; Gait speed → *n* = 71; Falls → *n* = 192; OMC → *n* = 263; Intracellular water → *n* = 163; Extracellular water → *n* = 163; Body water → *n* = 163; Bodyfat → *n* = 163; Appendicular Lean Soft Tissue → *n* = 163; Appendicular Lean Soft Tissue Index → *n* = 163; Length of Stay → *n* = 276; Early Warning Score → *n* = 283; Time to Readmission → *n* = 78; Prior Hospitalization → *n* = 282; Hemoglobin → *n* = 283; Creatinine → *n* = 283; Albumin → *n* = 282; eGFR → *n* = 283; Lactate Dehydrogenase → *n* = 221; Glucose → *n* = 277; C-reactive protein → *n* = 280; Living conditions → *n* = 243; Place after Discharge → *n* = 276; Barthel Index → *n* = 64; GDF-15 → *n* = 277; IL-6 → *n* = 277; IL-10 → *n* = 277; TNF-a → *n* = 277; TGF-b1 → *n* = 277

Patients < 80 years; *Men*; BMI → *n* = 236; HGS → *n* = 298; 30CST → *n* = 170; Gait speed → *n* = 31; Falls → *n* = 234; OMC → *n* = 281; Intracellular water → *n* = 192; Extracellular water → *n* = 192; Body water → *n* = 192; Bodyfat → *n* = 192; Appendicular Lean Soft Tissue → *n* = 192; Appendicular Lean Soft Tissue Index → *n* = 192; Length of Stay → *n* = 304; Early Warning Score → *n* = 312; Time to Readmission → *n* = 120; Prior Hospitalization → *n* = 308; Hemoglobin → *n* = 312; Creatinine → *n* = 309; Albumin → *n* = 310; eGFR → *n* = 310; Lactate Dehydrogenase → *n* = 216; Glucose → *n* = 300; C-reactive protein → *n* = 308; Living conditions → *n* = 298; Place after Discharge → *n* = 306; Barthel Index → *n* = 26; GDF-15 → *n* = 303; IL-6 → *n* = 303; IL-10 → *n* = 303; TNF-a → *n* = 303; TGF-b1 → *n* = 303

Women; BMI → *n* = 221; HGS → *n* = 274; 30CST → *n* = 165; Gait speed → *n* = 39; Falls → *n* = 214; OMC → *n* = 268; Intracellular water → *n* = 190; Extracellular water → *n* = 190; Body water → *n* = 190; Bodyfat → *n* = 190; Appendicular Lean Soft Tissue → *n* = 190; Appendicular Lean Soft Tissue Index → *n* = 190; Length of Stay → *n* = 276; Time to Readmission → *n* = 87; Prior Hospitalization → *n* = 281; Creatinine → *n* = 283; Albumin → *n* = 278; eGFR → *n* = 283; Lactate Dehydrogenase → *n* = 211; Glucose → *n* = 271; C-reactive protein → *n* = 282; Living conditions → *n* = 270; Place after Discharge → *n* = 276; Barthel Index → *n* = 36; GDF-15 → *n* = 271; IL-6 → *n* = 271; IL-10 → *n* = 271; TNF-a → *n* = 271; TGF-b1 → *n* = 271

Patients below 80 years of age had a significantly higher sample of men, while living conditions, place of discharge, and place after discharge were also significantly different between groups. It is worth noting that those 80 years and above had a higher prevalence of discharges to nursing homes and had a higher proportion of inpatients admitted to geriatric department compared to those below 80 years. All-cause death was significantly higher in those 80 years and above, they had higher mean Charlson Comorbidity Index, and a lower Early Warning Score. In addition, those below 80 years had higher body, intracellular, and extracellular water, leg lean soft tissue, arm lean soft tissue, ALST, ALSTI, HGS, 30CST, and OMC score compared to those 80 and above, while having lower CFS score. Moreover, adults below 80 years had higher levels of hemoglobin, eGFR, and TGF-b1, and lower levels of creatinine and GDF-15 vs. those ≥ 80 years.

## Association between longer LoS and sarcopenia indices

In men 80 years and above, no association was found between longer LoS and ALSTI, ALST, of HGS. Longer LoS was associated with lower gait speed in the unadjusted (b = -0.22, 95%CI -0.36 – -0.08, *p* < 0.01) and the fully adjusted model (b = -0.23, 95%CI -0.38 – -0.08, *p* < 0.01). Similar results were shown in relation to 30CST (Unadjusted: b = -4.07, 95%CI -5.99 – -2.15, *p* < 0.01; Model 2: b = -3.91, 95%CI -5.83 – -1.99, *p* < 0.01). Similar findings were found in women, for both gait speed and 30CST, in all models of associations (Gait speed: Model 2: b = -0.12, 95%CI -0.23 – -0.01, *p* = 0.04; 30CST: Model 2: b = -2.59, 95%CI -4.09 – -1.09, *p* < 0.01) (Table 3).

**Table 3:** Adjusted linear model associations for longer length of stay with sarcopenia indices in patients ≥ 80 and < 80 years of age

Age groups	LoS	Sex	Outcomes	Models	b (95%CI)	*p*	Sex	Outcomes	Models	b (95%CI)	*p*
Patients ≥ 80 years	Longer LoS	Men	ALSTI (kg/m^2^)	Unadjusted	-0.05 (-0.54–0.45)	0.86	Women	ALSTI (kg/m^2^)	Unadjusted	0.19 (-0.18–0.55)	0.31
				Model 1	0.09 (-0.34–0.51)	0.68			Model 1	0.14 (-0.15–0.42)	0.34
				Model 2	0.06 (-0.36–0.49)	0.77			Model 2	0.15 (-0.13–0.44)	0.29
			ALST (kg)	Unadjusted	1.20 (-0.67–3.07)	0.21		ALST (kg)	Unadjusted	0.66 (-0.40–1.72)	0.22
				Model 1	1.63 (-0.11–3.37)	0.07			Model 1	0.48 (-0.41–1.38)	0.29
				Model 2	1.54 (-0.20–3.28)	0.08			Model 2	0.51 (-0.39–1.40)	0.27
			HGS (kg)	Unadjusted	-2.60 (-5.26–0.06)	0.06		HGS (kg)	Unadjusted	-1.18 (-2.64–0.28)	0.11
				Model 1	-1.89 (-4.41–0.63	0.14			Model 1	-0.83 (-2.20–0.54)	0.23
				Model 2	-1.90 (-4.40–0.59)	0.13			Model 2	-0.77 (-2.14–0.60)	0.27
			Gait speed (m/s)	Unadjusted	-0.22 (-0.36 – -0.08)	< 0.01*		Gait speed (m/s)	Unadjusted	-0.13 (-0.24 – -0.01)	0.03*
				Model 1	-0.23 (-0.38 – -0.09)	< 0.01*			Model 1	-0.13 (-0.24 – -0.02)	0.03*
				Model 2	-0.23 (-0.38 – -0.08)	< 0.01*			Model 2	-0.12 (-0.23–0.01)	0.04*
			30 CST (repetitions)	Unadjusted	-4.07 (-5.99 – -2.15)	< 0.01*		30 CST (repetitions)	Unadjusted	-2.63 (-4.13 – -1.13)	< 0.01*
				Model 1	-3.93 (-5.84 – -2.02)	< 0.01*			Model 1	-2.64 (-4.14 – -1.14)	< 0.01*
				Model 2	-3.91 (-5.83 – -1.99)	< 0.01*			Model 2	-2.59 (-4.09 – -1.09)	< 0.01*
Patients < 80 years	Longer LoS	Men	ALSTI (kg/m^2^)	Unadjusted	-0.21 (-0.56–0.15)	0.26	Women	ALSTI (kg/m^2^)	Unadjusted	-0.002 (-0.37–0.37)	0.99
				Model 1	0.04 (-0.23–0.31)	0.77			Model 1	0.02 (-0.26–0.29)	0.90
				Model 2	0.03 (-0.23–0.30)	0.80			Model 2	0.02 (-0.26–0.30)	0.90
			ALST (kg)	Unadjusted	-0.62 (-1.92–0.68)	0.35		ALST (kg)	Unadjusted	-0.21 (-1.32–0.90)	0.71
				Model 1	0.14 (-0.93–1.20)	0.80			Model 1	-0.03 (-0.91–0.84)	0.94
				Model 2	0.07 (-0.99–1.14)	0.89			Model 2	-0.03 (-0.91–0.85)	0.95
			HGS (kg)	Unadjusted	-4.43 (-6.72 – -2.14)	< 0.01*		HGS (kg)	Unadjusted	-2.35 (-3.88 – -0.83)	< 0.01*
				Model 1	-3.67 (-5.90 – -1.43)	< 0.01*			Model 1	-1.70 (-3.20 – -0.20)	0.03*
				Model 2	-3.48 (-5.68 – -1.28)	< 0.01*			Model 2	-1.72 (-3.22 – -0.22)	0.03*
			Gait speed (m/s)	Unadjusted	-0.03 (-0.25–0.20)	0.80		Gait speed (m/s)	Unadjusted	-0.02 (-0.19–0.15)	0.82
				Model 1	0.01 (-0.22–0.23)	0.94			Model 1	-0.02 (-0.19–0.16)	0.86
				Model 2	-0.02 (-0.24–0.21)	0.87			Model 2	-0.03 (-0.20–0.15)	0.75
			30 CST (repetitions)	Unadjusted	-2.97 (-4.69 – -1.24)	< 0.01*		30 CST (repetitions)	Unadjusted	-1.69 (-3.39–0.02)	0.05
				Model 1	-3.17 (-4.87 – -1.48)	< 0.01*			Model 1	-1.36 (-3.07–0.35)	0.12
				Model 2	-3.08 (-4.75 – -1.41)	< 0.01*			Model 2	-1.36 (-3.07–0.35)	0.12

*Indicates significance; *p* < 0.05

30CST, 30-second chair stand test; ALST, appendicular lean soft tissue; HGS, handgrip strength; LoS, length of stay; ALSTI, appendicular lean soft tissue index

Model 1 adjusted for age and body mass index

Model 2 adjusted for Model 1 in addition to Charlson Comorbidity Index

In men < 80 years, no links were found between longer LoS and ALSTI, ALST, or gait speed. A negative association was found with HGS (Unadjusted: b = -4.43, 95%CI -6.72 – -2.14, *p* < 0.01; Model 2: b = -3.48, 95%CI -5.68 – -1.28, *p* < 0.01) and 30CST (Unadjusted: b = -2.97, 95%CI -4.69 – -1.24, *p* < 0.01; Model 2: b = -3.08, 95%CI -4.75 – -1.41, *p* < 0.01) in all models. In women < 80 years, these associations were common only for HGS (Unadjusted: b = -2.35, 95%CI -3.88 – -0.83, *p* < 0.01; Model 2: b = -1.72, 95%CI -3.22 – -0.22, *p* = 0.03) (Table 3).

When a logistic regression model was applied, in men ≥ 80 years, lower odds were depicted in terms of longer LoS and higher 30CST across all models (Model 2: OR = 0.24, 95%CI 0.09–0.66, *p* < 0.01). In women, although these links persisted for higher 30CST (Model 2: OR = 0.35, 95%CI 0.16–0.78, *p* < 0.01), lower odds of higher HGS (Model 2: OR = 0.55, 95%CI 0.31–0.99, *p* = 0.047) and faster gait speed (Model 2: OR = 0.12, 95%CI 0.03–0.53, *p* < 0.01) were also found (Table 4).

**Table 4:** Adjusted logistic model regressions between longer length of stay with sarcopenia indices in patients ≥ 80 and < 80 years of age. Data were calculated based on cut-offs employed by EWGSOP2

Age groups	LoS	Sex	Outcomes	Models	OR (95%CI)	*p*	Sex	Outcomes	Models	OR (95%CI)	*p*
Patients ≥ 80 years	Longer LoS	Men	Higher ALSTI	Unadjusted	1.00 (0.42–2.36)	1.00	Women	Higher ALSTI	Unadjusted	1.12 (0.60–2.10)	0.73
				Model 1	1.14 (0.44–3.00)	0.79			Model 1	1.18 (0.52–2.64)	0.70
				Model 2	1.03 (0.38–2.81)	0.95			Model 2	1.23 (0.54–2.78)	0.63
			Higher ALST	Unadjusted	2.19 (0.85–5.66)	0.11		Higher ALST	Unadjusted	1.58 (0.84–2.98)	0.16
				Model 1	2.83 (0.996–8.04)	0.05			Model 1	1.60 (0.78–3.27)	0.20
				Model 2	2.72 (0.91–8.08)	0.07			Model 2	1.62 (0.79–3.34)	0.19
			Higher HGS	Unadjusted	0.54 (0.26–1.11)	0.09		Higher HGS	Unadjusted	0.53 (0.30–0.92)	0.02*
				Model 1	0.62 (0.29–1.35)	0.23			Model 1	0.55 (0.31–1.00)	0.049*
				Model 2	0.61 (0.28–1.34)	0.22			Model 2	0.55 (0.31–0.99)	0.047*
			Faster gait speed	Unadjusted	0.33 (0.09–1.28)	0.11		Faster gait speed	Unadjusted	0.14 (0.04–0.56)	< 0.01*
				Model 1	0.27 (0.06–1.13)	0.07			Model 1	0.12 (0.03–0.51)	< 0.01*
				Model 2	0.26 (0.06–1.15)	0.08			Model 2	0.12 (0.03–0.53)	< 0.01*
			Higher 30CST	Unadjusted	0.24 (0.09–0.63)	< 0.01*		Higher 30CST	Unadjusted	0.35 (0.16–0.78)	0.01*
				Model 1	0.24 (0.09–0.66)	< 0.01*			Model 1	0.35 (0.16–0.77)	0.01*
				Model 2	0.24 (0.09–0.66)	< 0.01*			Model 2	0.35 (0.16–0.78)	0.01*
			Sarcopenia (with low HGS)	Unadjusted	1.24 (0.50–3.08)	0.64		Sarcopenia (with low HGS)	Unadjusted	0.93 (0.46–1.87)	0.84
				Model 1	1.11 (0.42–2.93)	0.83			Model 1	0.94 (0.41–2.14)	0.88
				Model 2	1.29 (0.46–3.57)	0.63			Model 2	0.93 (0.41–2.13)	0.87
			Sarcopenia (with low 30CST)	Unadjusted	1.73 (0.64–4.72)	0.28		Sarcopenia (with low 30CST)	Unadjusted	1.45 (0.68–3.07)	0.34
				Model 1	1.64 (0.55–4.87)	0.37			Model 1	1.78 (0.72–4.47)	0.21
				Model 2	1.68 (0.56–5.02)	0.35			Model 2	1.79 (0.72–4.46)	0.21
Patients < 80 years	Longer LoS	Men	Higher ALSTI	Unadjusted	0.55 (0.30–1.01)	0.05	Women	Higher ALSTI	Unadjusted	1.20 (0.62–2.32)	0.60
				Model 1	0.79 (0.36–1.74)	0.56			Model 1	1.22 (0.52–2.91)	0.65
				Model 2	0.74 (0.33–1.66)	0.47			Model 2	1.23 (0.52–2.91)	0.65
			Higher ALST	Unadjusted	0.60 (0.31–1.16)	0.13		Higher ALST	Unadjusted	0.99 (0.55–1.79)	0.97
				Model 1	0.83 (0.38–1.81)	0.64			Model 1	1.03 (0.49–2.15)	0.95
				Model 2	0.82 (0.38–1.80)	0.62			Model 2	1.03 (0.49–2.16)	0.94
			Higher HGS	Unadjusted	0.45 (0.26–0.78)	< 0.01*		Higher HGS	Unadjusted	0.50 (0.27–0.92)	0.03*
				Model 1	0.49 (0.28–0.86)	0.01*			Model 1	0.60 (0.31–1.15)	0.12
				Model 2	0.50 (0.28–0.89)	0.02*			Model 2	0.60 (0.31–1.15)	0.12
			Faster gait speed	Unadjusted	0.62 (0.14–2.82)	0.53		Faster gait speed	Unadjusted	1.75 (0.37–8.33)	0.48
				Model 1	0.44 (0.06–3.43)	0.44			Model 1	1.78 (0.36–8.81)	0.48
				Model 2	0.48 (0.06–4.00)	0.50			Model 2	1.72 (0.34–8.57)	0.51
			Higher 30CST	Unadjusted	0.44 (0.23–0.85)	0.02*		Higher 30CST	Unadjusted	0.59 (0.29–1.17)	0.13
				Model 1	0.38 (0.19–0.75)	< 0.01*			Model 1	0.61 (0.31–1.24)	0.17
				Model 2	0.38 (0.19–0.78)	< 0.01*			Model 2	0.61 (0.30–1.24)	0.17
			Sarcopenia (with low HGS)	Unadjusted	1.41 (0.68–2.95)	0.36		Sarcopenia (with low HGS)	Unadjusted	1.27 (0.50–3.22)	0.61
				Model 1	0.90 (0.39–2.11)	0.90			Model 1	1.47 (0.51–4.23)	0.47
				Model 2	0.92 (0.39–2.16)	0.85			Model 2	1.50 (0.52–4.31)	0.45
			Sarcopenia (with low 30CST)	Unadjusted	3.02 (1.29–7.08)	0.01*		Sarcopenia (with low 30CST)	Unadjusted	0.80 (0.33–1.97)	0.63
				Model 1	2.48 (0.86–7.20)	0.10			Model 1	0.68 (0.23–2.00)	0.48
				Model 2	2.66 (0.90–7.87)	0.08			Model 2	0.67 (0.22–1.99)	0.47

*Indicates significance; *p* < 0.05

30CST, 30-second chair stand test; ALST, appendicular lean soft tissue; HGS, handgrip strength; LoS, length of stay; ALSTI, appendicular lean soft tissue index

Model 1 adjusted for age and body mass index

Model 2 adjusted for Model 1 in addition to Charlson Comorbidity Index

In men < 80 years, longer LoS was associated with lower odds of higher HGS (Model 2: OR = 0.50, 95%CI 0.29–0.89, *p* = 0.02) and higher 30CST (Model 2: OR = 0.38, 95%CI 0.19–0.78, *p* < 0.01). Higher odds of sarcopenia (with low 30CST) were also shown, however, only in the unadjusted model (OR 3.02, 95%CI 1.29–7.08, *p* = 0.01). In women, the only (negative) association was found was pertinent to longer LoS with higher HGS in the unadjusted model (OR = 0.50, 95%CI 0.27–0.92, *p* = 0.03) (Table 4).

## Association between sarcopenia indices with LoS

In men patients ≥ 80 years, only higher 30CST was associated with shorter LoS duration (Unadjusted: b = -4.58, 95%CI -7.93 – -1.24, *p* < 0.01; Model 2: b = -4.02, 95%CI -7.47 – -0.58, *p* = 0.02). No links were found between higher ALSTI, ALST, HGS, or faster gait speed with LoS duration. On the contrary, in women patients of this age group, higher HGS (Model 2: b = -2.18, 95%CI -4.07 – -0.30, *p* = 0.02), faster gait speed (Model 2: b = -5.12, 95%CI -9.70 – -0.55, *p* = 0.03), and higher 30CST repetitions (Model 2: b = -3.91, 95%CI -6.50 – -1.31, *p* < 0.01) were associated with shorter LoS duration across all models (Table 5).

**Table 5:** Adjusted linear model associations between sarcopenia indices based on cut-offs employed by EWGSOP2 with length of stay in patients ≥ 80 and < 80 years of age

Outcomes	Sex	Patients ≥ 80 years			Patients < 80 years		
		LoS			LoS		
		Unadjusted	Model 1	Model 2	Unadjusted	Model 1	Model 2
		b (95%CI); p			b (95%CI); p		
Higher ALSTI (kg/m^2^)	Men	-2.10 (-6.00–1.81); 0.29	-0.78 (-4.99–3.43); 0.71	-1.05 (-5.40–3.30); 0.63	-0.16 (-2.52–2.19); 0,89	-0.39 (-3.25–2.48), 0.79	-0.56 (-3.42–2.31); 0.70
	Women	0.67 (-0.22–1.55); 0.14	1.14 (-0.01–2.28); 0.05	1.09 (-0.06–2.25); 0.06	0.53 (-0.15–1.21); 0.13	0.81 (-0.11–1.73); 0.08	0.80 (-0.11–1.72); 0.09
Higher ALST (kg)	Men	1.80 (-2.39–5.99); 0.40	3.81 (-0.50–8.11); 0.08	3.84 (-0.67–8.36); 0.09	-1.53 (-4.06–0.99); 0.23	-1.97 (-4.84–0.90); 0.18	-1.99 (-4.85–0.87); 0.17
	Women	1.15 (-0.95–3.26); 0.28	1.43 (-0.95–3.80); 0.24	1.39 (-0.99–3.77); 0.25	0.66 (-1.12–2.45); 0.46	0.57 (-1.58–2.73); 0.60	0.60 (-1.55–2.74); 0.59
Higher HGS (kg)	Men	-1.69 (-4.56–1.18); 0.25	-0.55 (-3.60–2.50); 0.72	-0.45 (-3.54–2.64); 0.78	-3.15 (-5.06 – -1.23); <0.01*	-3.29 (-5.29 – -1.28); <0.01*	-3.18 (-5.21 – -1.16); <0.01*
	Women	-2.32 (-4.12 – -0.52); 0.01*	-2.19 (-4.07 – -0.31); 0.02*	-2.18 (-4.07 – -0.30); 0.02*	-2.29 (-4.31 – -0.28); 0.03*	-1.51 (-3.62–0.60); 0.16	-1.51 (-3.62–0.60); 0.16
Faster Gait speed (m/s)	Men	-3.41 (-7.76–0.94); 0.12	-3.66 (-8.00–0.67); 0.10	-4.23 (-8.58–0.11); 0.06	-2.12 (-5.27–1.03); 0.18	-1.37 (-4.43–1.69); 0.37	-2.31 (-5.50–0.88); 0.15
	Women	-4.77 (-9.16 – -0.38); 0.03*	-4.94 (-9.50 – -0.38); 0.03*	-5.12 (-9.70 – -0.55); 0.03*	0.97 (-4.19–6.12); 0.71	1.38 (-3.91–6.66); 0.60	1.36 (-4.02–6.74); 0.61
Higher 30CST (repetitions)	Men	-4.58 (-7.93 – -1.24); <0.01*	-4.11 (-7.53 – -0.68); 0.02*	-4.02 (-7.47 – -0.58); 0.02*	-2.88 (-4.89 – -0.87); <0.01*	-3.41 (-5.46 – -1.37); <0.01*	-3.49 (-5.58 – -1.41), < 0.01*
	Women	-3.71 (-6.29 – -1.14); <0.01*	-3.86 (-6.44 – -1.27); <0.01*	-3.91 (-6.50 – -1.31); <0.01*	-1.82 (-4.24–0.59); 0.14	-1.91 (-4.26–0.44); 0.11	-1.92 (-4.27–0.43); 0.11

*Indicates significance; *p* < 0.05

30CST, 30-second chair stand test; ALST, appendicular lean soft tissue; HGS, handgrip strength; LoS, length of stay; ALSTI, appendicular lean soft tissue index

Model 1 adjusted for age and body mass index

Model 2 adjusted for Model 1 in addition to Charlson Comorbidity Index

In men < 80 years, higher HGS and 30CST were associated with shorter LoS (Higher HGS: Model 2: b = -3.18, 95%CI -5.21 – -1.16, *p* < 0.01; Higher 30CST: Model 2: b = -3.49, 95%CI -5.58 – -1.41, *p* < 0.01). In women, higher HGS was linked to shorted LoS duration only in the unadjusted model (b = -2.29, 95%CI -4.31 – -0.28, *p* = 0.03). No other links were found among sarcopenia indices with LoS (Table 5).

Using a logistic regression, in men ≥ 80 years, higher ALST was associated with increased odds of having a longer LoS in the adjusted models (Model 2: OR = 3.24, 95%CI 1.07–9.82, *p* = 0.04). Lower odds of longer LoS were presented for higher 30CST across all models (Model 2: OR = 0.23, 95%CI 0.09–0.64, *p* < 0.01). No links were found in relation to higher ALSTI, HGS, body water, faster gait speed, or sarcopenia. In women, higher HGS, 30CST, and faster gait speed were linked to lower odds of longer LoS across models (Higher HGS: Model 2: OR = 0.54, 95%CI 0.30–0.98, *p* = 0.04; Faster gait speed: Model 2: OR = 0.12, 95%CI 0.03–0.53, *p* < 0.01; Higher 30CST: Model 2: OR = 0.35, 95%CI 0.16–0.78, *p* = 0.01) (Table S1).

In men < 80 years of age, higher HGS and 30CST were linked to lower odds of longer LoS in each model (Higher HGS: Model 2: OR = 0.50, 95%CI 0.28–0.89, *p* = 0.02; Higher 30CST: Model 2: OR = 0.38, 95%CI 0.18–0.76, *p* < 0.01). In addition, sarcopenia (using low 30CST) was associated with greater odds of longer LoS (OR = 3.14, 95%CI 1.16–8.54, *p* = 0.03). Women < 80 years with higher HGS had lower odds of longer LoS only in the unadjusted model (OR = 0.50, 95%CI 0.27–0.92, *p* = 0.03). No other links were shown when the rest of sarcopenia indices and body water were used in the analysis (Table S1).

## Discussion

This subset cohort of the PROTECT study highlights significant differences in clinical characteristics and outcomes between patients aged < 80 years and those aged ≥ 80 years, with notable implications for hospital LoS and sarcopenia indices. These results provide insights into the relationship among age, muscle mass, strength, and functional status, and hospitalization outcomes, particularly in the context of risk-stratification, geriatric care, rehabilitation, and sarcopenia management. Older patients (≥ 80 years) were more frequently discharged to nursing homes and admitted to geriatric departments, reflecting their increased frailty and dependency, which was depicted via their elevated CFS and CCI, and lower EWS scores. Younger patients (< 80 years) demonstrated superior physical, functional, and cognitive parameters, as well as biochemical characteristics such as higher hemoglobin, eGFR, and TGF-β1 levels, and lower creatinine and GDF-15 levels.

The associations between sarcopenia indices and LoS varied distinctly by age and sex, revealing key insights into the role of functional performance in hospitalization outcomes. In men aged ≥ 80 years, longer LoS was associated with lower gait speed and reduced 30CST result, but not with ALSTI, ALST, or HGS. Similarly, in women ≥ 80 years, longer LoS was linked to lower gait speed and 30CST scores, with HGS also showing a negative association in adjusted models. The 30CST, a robust indicator of lower body strength and endurance, was consistently linked to shorter LoS in both sexes within this age group, aligning with its established role as a surrogate for physical function and exercise tolerance in geriatric populations (Takeda et al., 2025; Kobayashi et al., 2024). The absence of associations between ALSTI, ALST, and hospitalization outcomes suggests that muscle mass alone may not have a strong link to these outcomes compared to muscle strength or physical function, supporting the emphasis on functional measures in sarcopenia assessment (Cruz-Jentoft et al., 2019). In patients aged < 80 years, HGS and 30CST were negatively associated with LoS in men across all models. In women, however, only HGS showed a significant association with LoS, and only in unadjusted models, suggesting weaker or less consistent links. These sex-specific differences may reflect variations in neuromuscular physiology [24], warranting further exploration to understand underlying mechanisms. Notably, sarcopenia (defined by low ALSTI and low HGS or 30CST) was associated with higher odds of longer LoS in men < 80 years, indicating that functional impairments could significantly impact recovery and healthcare resource utilization, even in relatively younger patients. Moreover, in men ≥ 80 years, higher 30CST scores were consistently linked to lower odds of prolonged LoS. However, higher ALST was unexpectedly associated with increased LoS odds in adjusted models, a counterintuitive finding that may reflect confounding factors, such as underlying comorbidities leading to extended hospitalization stay despite higher ALST or increased oedema, altering BIA measurements in relation to ALST. In women ≥ 80 years, higher HGS, faster gait speed, and greater 30CST scores were all associated with reduced odds of longer LoS, highlighting the potentially protective effect of functional capacity in this group. Additionally, among patients < 80 years, men with higher HGS and 30CST scores had lower odds of prolonged LoS, while women showed a weaker association, limited to HGS in unadjusted models and pointing to other influencing factors, such as sex-specific neuromuscular sex-specific implications [25]. Longitudinal studies would be critical to assess whether sarcopenia could reflect longer LoS, that may determine whether routine assessment of muscle strength and physical function at hospital admission could serve as a tool for identifying high-risk patients. Subsequently, this could enable targeted interventions to optimize recovery and reduce hospital stay durations, particularly in those with projected long LoS.

### Clinical implications and future directions

These findings may help support the integration of cheap and time effective functional assessments that require minimal training in clinical practice, such as 30CST and HGS, which could guide risk stratification for longer LoS and rehabilitation strategies. Exercise programs targeting lower extremity strength and mobility, such as resistance training or balance-focused interventions, may be particularly effective in improving functional capacity, potentially reducing LoS and enhancing recovery [26]. Future research should focus on longitudinal studies to generate more robust data and establish causal relationships between functional measures and LoS in geriatric populations. Randomized controlled trials evaluating targeted interventions (i.e., structured exercise programs, dietary interventions, or combined approaches), could further clarify their impact on LoS and functional outcomes, taking into account sex-specific factors, such as hormonal differences.

### Limitations

This study has several limitations that should be considered when interpreting the findings. First, this work was based on cross-sectional data, which precludes causal inferences. In addition, the reliance on specific sarcopenia indices may also overlook other relevant parameters, such as muscle quality or nutritional status, which were not used as confounders in these associations. Furthermore, the weaker associations in women < 80 years suggest potentially unexamined psychosocial or environmental factors compared to men, that warrant further investigation. Added to this, it is worth noting that considering the separation of multiple groups, for age- and sex-specific links, the sample size was generally limited. A further limitation is the large number of regressions performed across multiple age groups, sex strata, outcomes, and adjustment models, raising concerns about multiple comparisons and increasing the risk of Type I error. Moreover, the diagnosis categories were grouped into broad diagnostic categories to ensure manageable analyses across numerous individual diagnoses. Consequently, some diagnostic nuances and heterogeneity within categories may not be fully captured, which may be a major determinant of both baseline functional status and LoS. Finally, the counterintuitive association between higher ALST and longer LoS in men ≥ 80 years highlights potential residual confounding, such as specific underlying comorbidities, that were not accounted for in adjusted models.

## Conclusions

This study presented cross-sectional links of functional performance with hospital LoS across age and sex groups. In patients ≥ 80 years, lower 30CST and slower gait speed scores were consistently linked to longer LoS, with HGS also associated in women. In those < 80 years, HGS and 30CST linked to shorter LoS in men, with weaker associations in women. Future longitudinal research is warranted to provide more robust relationship and confirm causality, which may highlight the potential utility of 30CST and HGS as cost-effective tools for risk stratification and rehabilitation planning in geriatric patients, particularly in those aged 80 years and above.

## Supplementary Information

Below is the link to the electronic supplementary material.

Supplementary Material 1

## Data Availability

No datasets were generated or analysed during the current study.
