# Supplementary material for: Associations of length of stay with sarcopenia parameters in geriatric patients: age and sex differences in the oldest old
Source: Aging Clin Exp Res. 2026 Jun 25;38(1):190. doi: 10.1007/s40520-026-03442-z (PMC13582360; doi:10.1007/s40520-026-03442-z)
Supplement: Supplementary file 1 [file 40520_2026_3442_MOESM1_ESM.docx]

**Table S1.** Adjusted logistic model regressions between sarcopenia indices based on cut-offs employed by EWGSOP2 with longer length of stay in patients ≥80 and <80 years of age.

| Outcomes | Sex | Patients ≥80 years | | | Patients <80 years | | |
| --- | --- | --- | --- | --- | --- | --- | --- |
|  |  | Unadjusted | Model 1 | Model 2 | Unadjusted | Model 1 | Model 2 |
|  |  | Longer LoS | | | Longer LoS | | |
|  |  | OR (95%CI); p | | | OR (95%CI); p | | |
| Higher ALSTI (kg/m^2^) | Men | 1.00 (0.42 – 2.36); 1.00 | 1.28 (0.49 – 3.36); 0.62 | 1.19 (0.44 – 3.21); 0.74 | 0.55 (0.30 – 1.01); 0.05 | 0.69 (0.33 – 1.46); 0.33 | 0.67 (0.31 – 1.41); 0.29 |
|  | Women | 1.12 (0.60 – 2.10); 0.73 | 0.99 (0.45 – 2.16); 0.97 | 1.02 (0.46 – 2.25); 0.96 | 1.20 (0.62 – 2.32); 0.60 | 1.34 (0.61 – 2.96); 0.47 | 1.34 (0.61 – 2.95); 0.47 |
| Higher ALST (kg) | Men | 2.19 (0.85 – 5.66); 0.11 | 3.30 (1.13 – 9.59); 0.03* | 3.24 (1.07 – 9.82); 0.04* | 0.60 (0.31 – 1.16); 0.13 | 0.72 (0.34 – 1.53); 0.39 | 0.72 (0.34 – 1.52); 0.39 |
|  | Women | 1.58 (0.84 – 2.98); 0.16 | 1.60 (0.78 – 3.27); 0.20 | 1.62 (0.79 – 3.33); 0.19 | 0.99 (0.55 – 1.79); 0.97 | 1.11 (0.54 – 2.28); 0.78 | 1.11 (0.54 – 2.27); 0.78 |
| Higher HGS (kg) | Men | 0.54 (0.26 – 1.11); 0.09 | 0.60 (0.28 – 1.30); 0.20 | 0.59 (0.27 – 1.29); 0.19 | 0.45 (0.26 – 0.78); <0.01* | 0.49 (0.28 – 0.86); 0.01* | 0.50 (0.28 – 0.89); 0.02* |
|  | Women | 0.53 (0.30 – 0.92); 0.02* | 0.55 (0.30 – 0.99); 0.046* | 0.54 (0.30 – 0.98); 0.04* | 0.50 (0.27 – 0.92); 0.03* | 0.60 (0.31 – 1.15); 0.12 | 0.60 (0.31 – 1.15); 0.12 |
| Faster Gait speed (m/s) | Men | 0.33 (0.09 – 1.28); 0.11 | 0.27 (0.06 – 1.14); 0.07 | 0.27 (0.06 – 1.16); 0.08 | 0.62 (0.14 – 2.82); 0.53 | 0.79 (0.13 – 4.78); 0.80 | 0.50 (0.07 – 3.75); 0.50 |
|  | Women | 0.14 (0.04 – 0.56); <0.01* | 0.12 (0.03 – 0.52); <0.01* | 0.12 (0.03 – 0.53); <0.01* | 1.75 (0.37 – 8.33); 0.48 | 1.78 (0.36 – 8.71); 0.48 | 1.74 (0.35 – 8.66); 0.50 |
| Higher 30CST (repetitions) | Men | 0.24 (0.09 – 0.63); <0.01* | 0.23 (0.09 – 0.64); <0.01* | 0.23 (0.09 – 0.64); <0.01* | 0.044 (0.23 – 0.85); 0.02* | 0.37 (0.19 – 0.75); <0.01* | 0.38 (0.18 – 0.76); <0.01* |
|  | Women | 0.35 (0.16 – 0.78); 0.01* | 0.35 (0.16 – 0.77); <0.01* | 0.35 (0.16 – 0.78); 0.01* | 0.59 (0.29 – 1.17); 0.13 | 0.61 (0.31 – 1.24); 0.17 | 0.61 (0.31 – 1.24); 0.17 |
| Sarcopenia (with low HGS) | Men | 1.24 (0.50 – 3.08); 0.64 | 1.02 (0.39 – 2.69); 0.97 | 1.14 (0.41 – 3.14); 0.80 | 1.41 (0.68 – 2.95); 0.36 | 1.08 (0.48 – 2.41); 0.86 | 1.09 (0.49 – 2.45); 0.83 |
|  | Women | 1.145 (0.68 – 3.07); 0.34 | 2.08 (0.84 – 5.15); 0.11 | 2.08 (0.84 – 5.15); 0.11 | 0.80 (0.33 – 1.97); 0.63 | 0.62 (0.22 – 1.72); 0.36 | 0.62 (0.22 – 1.72); 0.36 |
| Sarcopenia (with low 30CST) | Men | 1.73 (0.64 – 4.72); 0.28 | 1.49 (0.51 – 4.39); 0.47 | 1.52 (0.51 – 4.52); 0.45 | 3.02 (1.29 – 7.08); 0.01* | 3.01 (1.12 – 8.09); 0.03* | 3.14 (1.16 – 8.54); 0.03* |
|  | Women | 0.93 (0.46 – 1.87); 0.84 | 1.10 (0.49 – 2.48); 0.81 | 1.10 (0.49 – 2.47); 0.82 | 1.27 (0.50 – 3.22); 0.61 | 1.22 (0.45 – 3.30); 0.70 | 1.23 (0.45 – 3.34); 0.69 |
| Higher bodywater (kg) | Men | 1.31 (0.57 – 2.99); 0.53 | 1.61 (0.66 – 3.94); 0.30 | 1.60 (0.65 – 3.92); 0.31 | 0.70 (0.39 – 1.24); 0.22 | 0.91 (0.47 – 1.75); 0.77 | 0.88 (0.46 – 1.71); 0.88 |
|  | Women | 1.67 (0.88 – 3.15); 0.12 | 1.66 (0.82 – 3.37); 0.16 | 1.72 (0.84 – 3.52); 0.14 | 1.11 (0.62 – 1.99); 0.73 | 1.31 (0.64 – 2.67); 0.46 | 1.31 (0.64 – 2.69); 0.46 |
| Higher intracellular water (kg) | Men | 1.43 (0.62 – 3.30); 0.40 | 1.81 (0.73 – 4.52); 0.20 | 1.81 (0.72 – 4.52); 0.21 | 0.70 (0.39 – 1.24); 0.22 | 0.93 (0.48 – 1.80); 0.82 | 0.92 (0.47 – 1.78); 0.80 |
|  | Women | 1.67 (0.88 – 3.17); 0.12 | 1.66 (0.81 – 3.42); 0.17 | 1.71 (0.83 – 3.54); 0.15 | 1.21 (0.67 – 2.18); 0.52 | 1.47 (0.72 – 3.01); 0.30 | 1.48 (0.72 – 3.03); 0.29 |
| Higher extracellular water (kg) | Men | 1.85 (0.81 – 4.23); 0.15 | 2.35 (0.93 – 5.97); 0.07 | 2.23 (0.91 – 5.90); 0.08 | 0.66 (0.37 – 1.16); 0.15 | 0.82 (0.43 – 1.55); 0.55 | 0.80 (0.42 – 1.52); 0.49 |
|  | Women | 1.58 (0.84 – 2.97); 0.16 | 1.59 (0.79 – 3.19); 0.20 | 1.61 (0.80 – 3.24); 0.18 | 1.15 (0.65 – 2.06); 0.63 | 1.29 (0.65 – 2.55); 0.46 | 1.30 (0.66 – 2.56); 0.46 |

*Indicates significance; *p* < 0.05.
30CST, 30-second chair stand test; ALST, appendicular lean soft tissue; HGS, handgrip strength; LoS, length of stay; ALSTI, appendicular lean soft tissue index
Model 1 adjusted for age and body mass index
Model 2 adjusted for Model 1 in addition to Charlson Comorbidity Index
